# Supplementary material for: Synthesis and Biological Evaluation of Novel Furopyridone Derivatives as Potent Cytotoxic Agents against Esophageal Cancer
Source: Int J Mol Sci. 2024 Sep 5;25(17):9634. doi: 10.3390/ijms25179634 (PMC11395057; doi:10.3390/ijms25179634)

Synthesis and Biological Evaluation of Novel Furopyidone Derivatives as Potent Cytotoxic Agents Against Esophageal Cancer

Xingyu Ren, Jiaojiao Zhang, Anying Dai, Pengzhi Sun, Yibo Zhang, Lu Jin and Le Pan *

College of Chemistry and Chemical Engineering, Xinjiang Agricultural University, Urumqi 830052, China; 18299520272@163.com (X.R.); koh9391421@163.com (J.Z.); 13609979146@163.com (A.D.); sun8302587@163.com (P.S.); 18290824750@163.com (Y.Z.); lu_lu_jin@163.com (L.J.)

* Correspondence: chempan03@163.com

Data of ¹H NMR, ¹³C NMR

¹H NMR of compound 2a

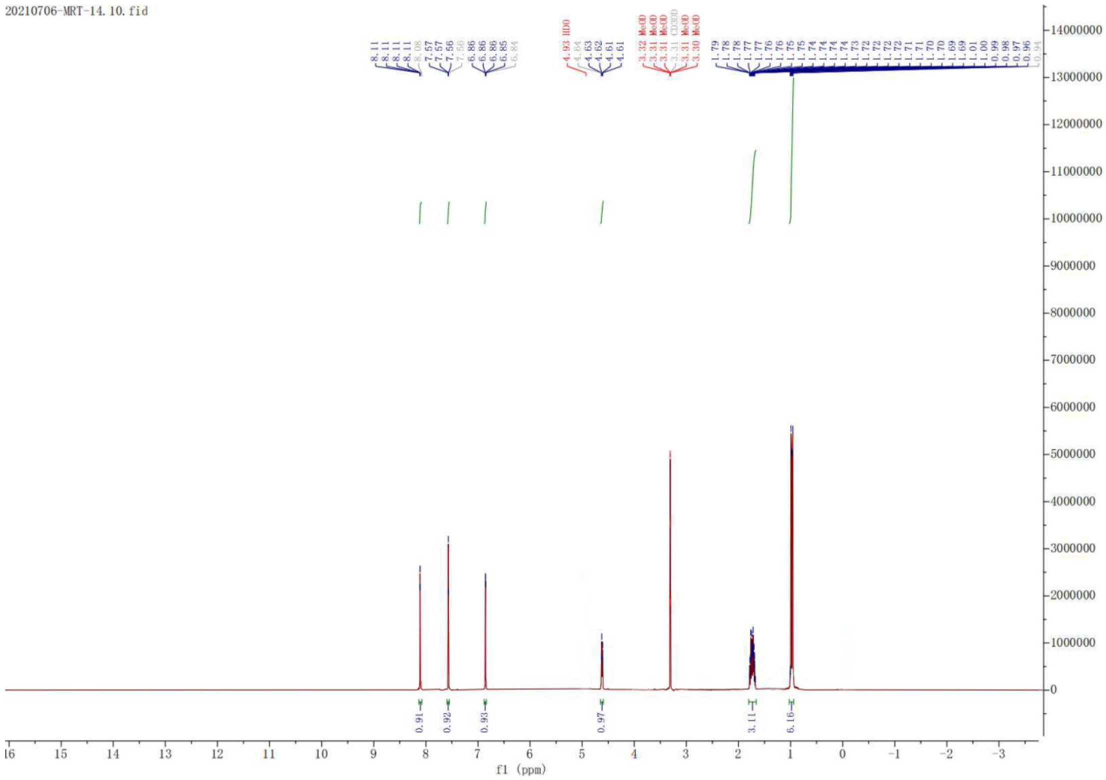

¹³C NMR of compound 2a

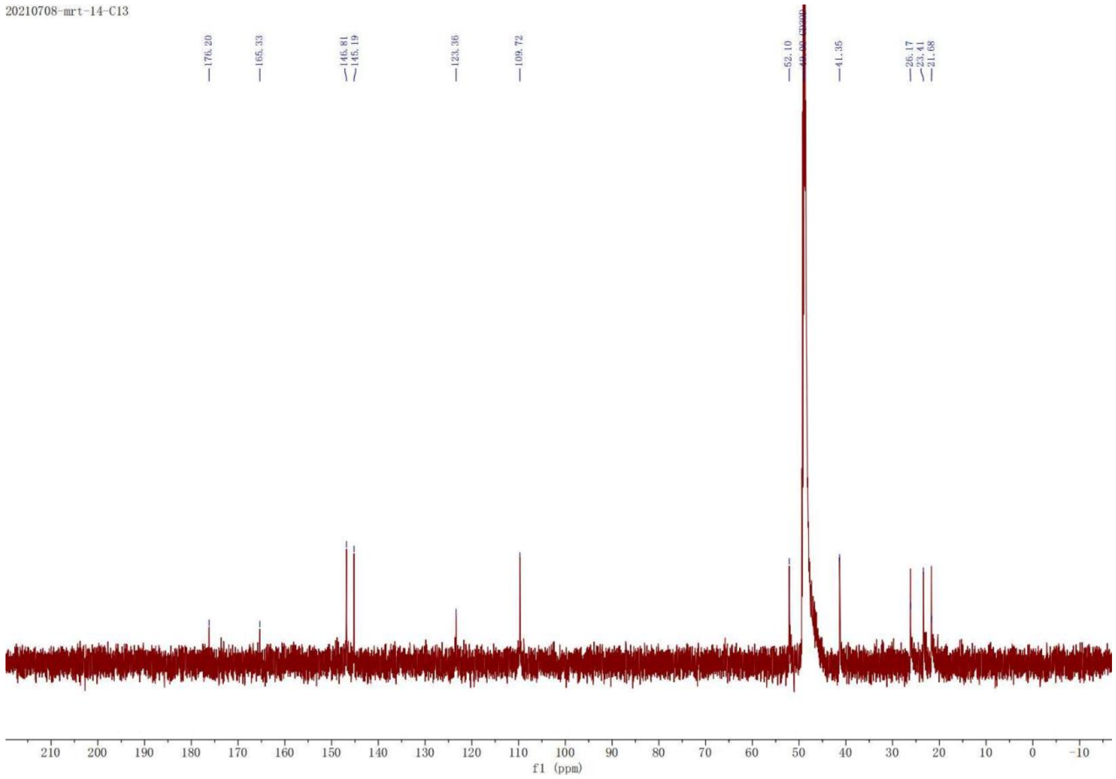

^{1}H NMR of compound 2b

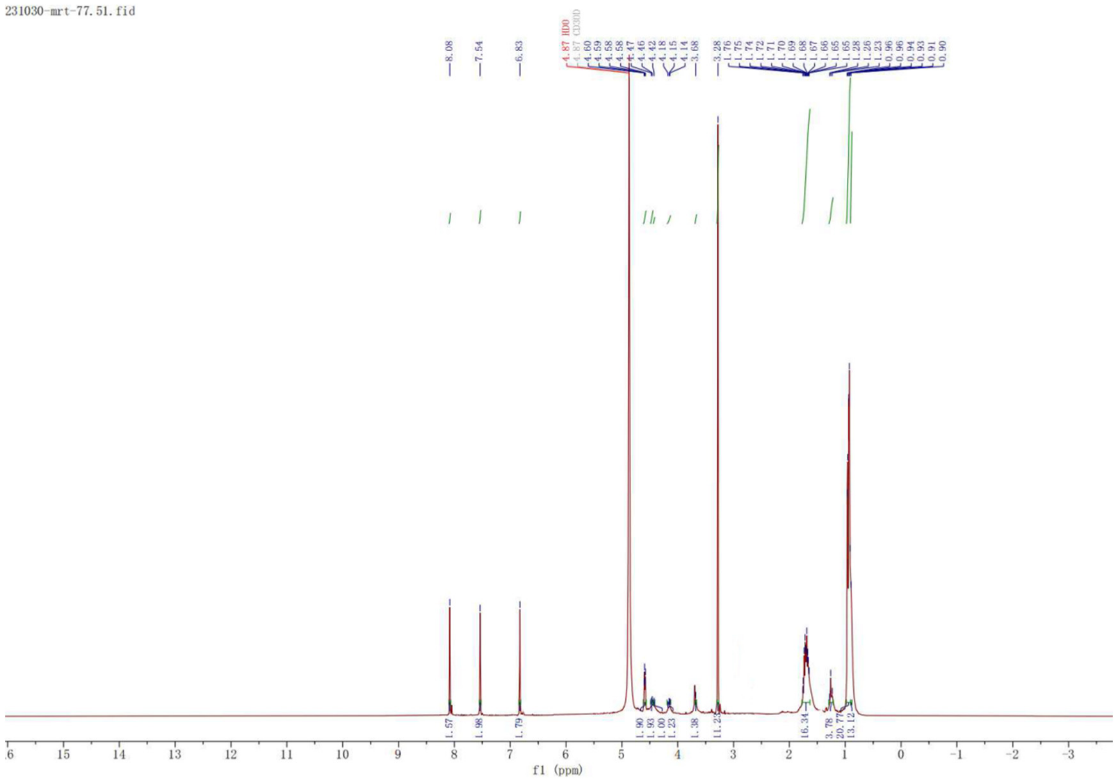

^{13}C NMR of compound 2b

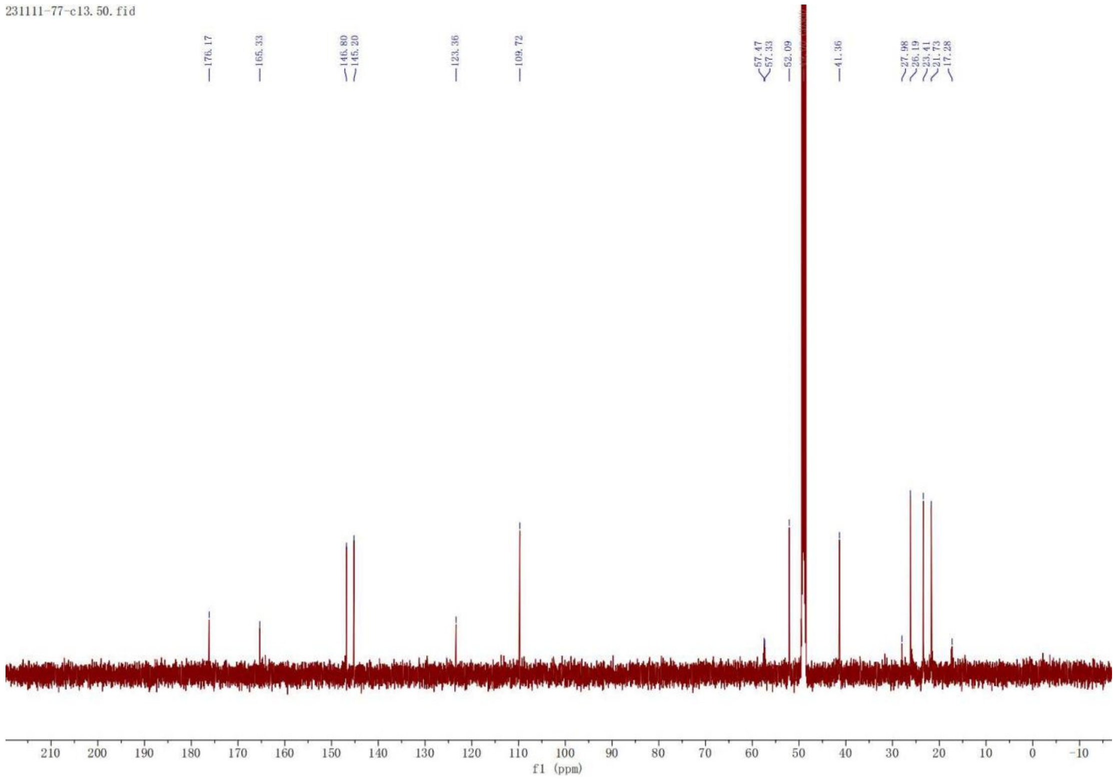

^{1}H NMR of compound 2c

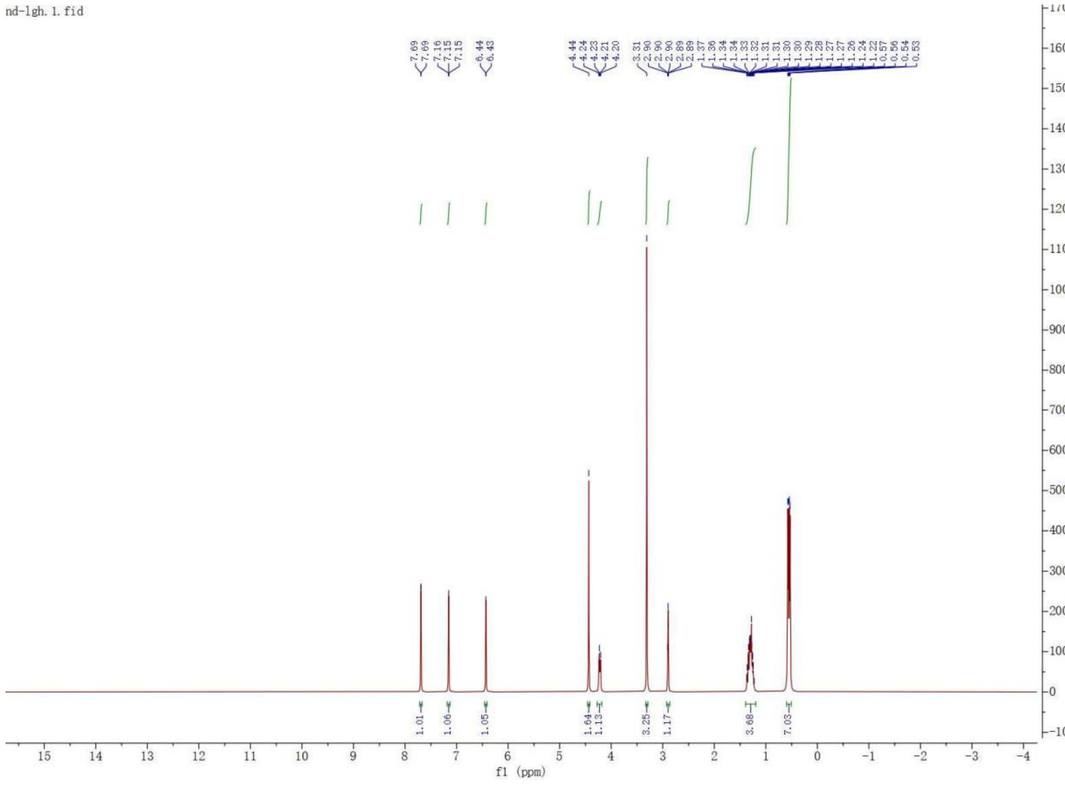

^{13}C NMR of compound 2c

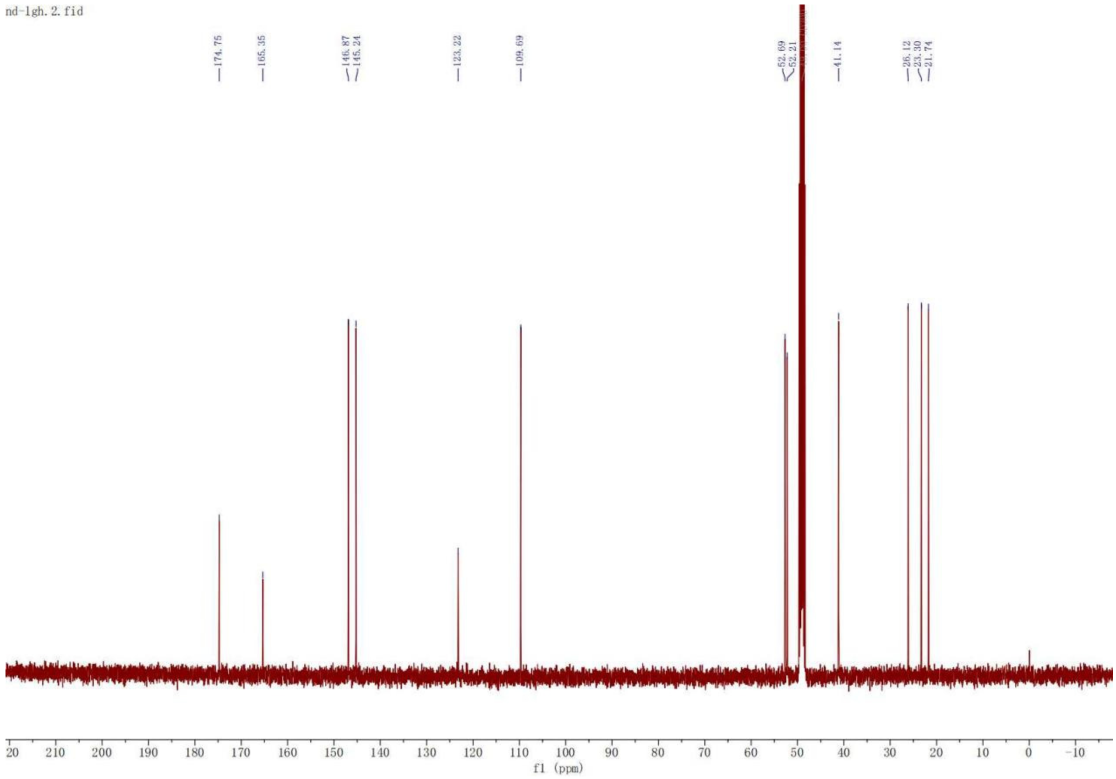

1H NMR of compound 2d

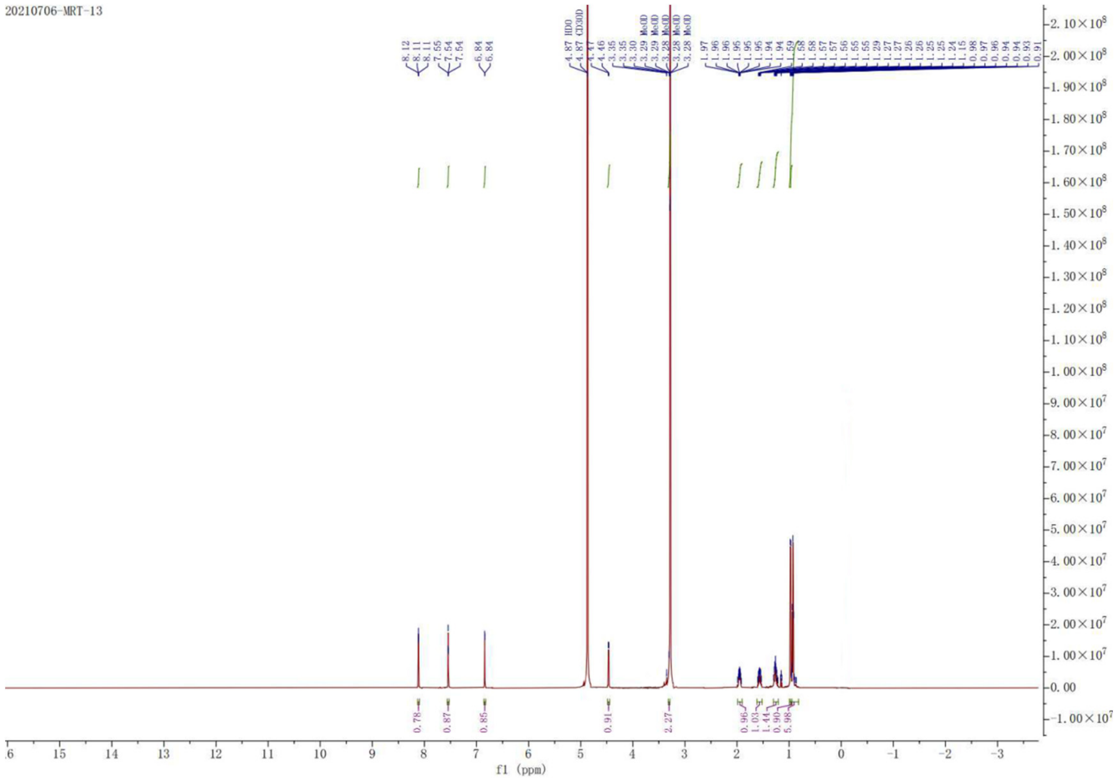

13C NMR of compound 2d

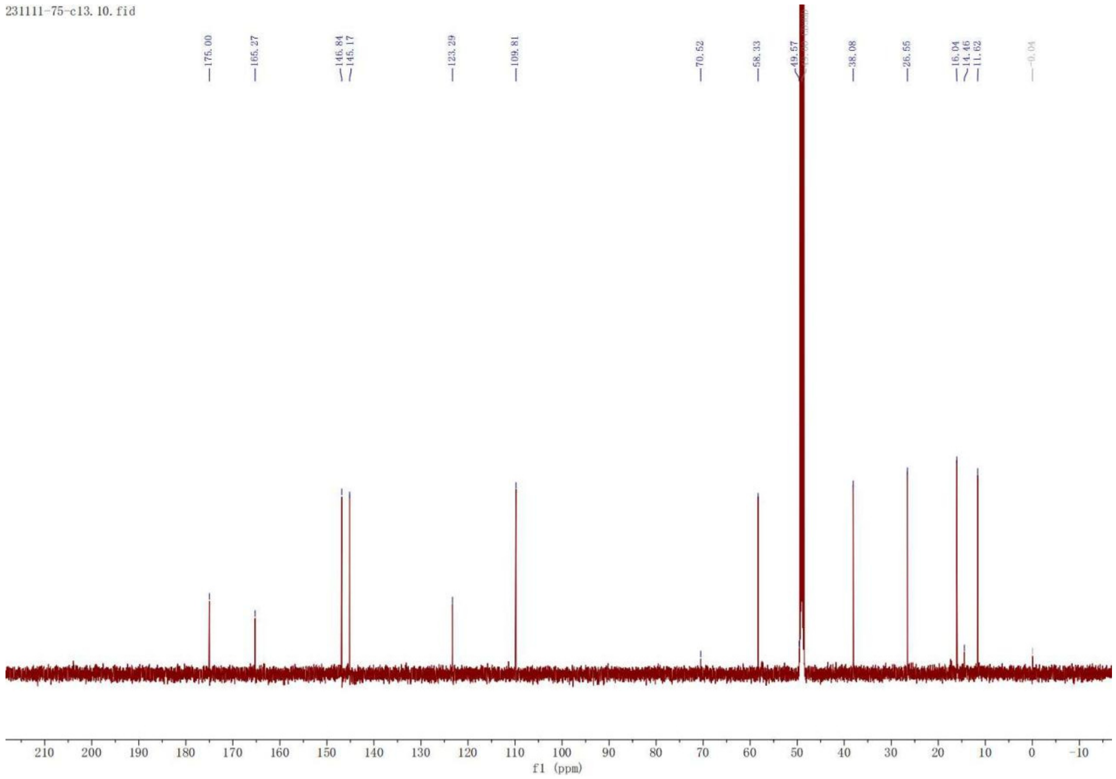

¹H NMR of compound 2e

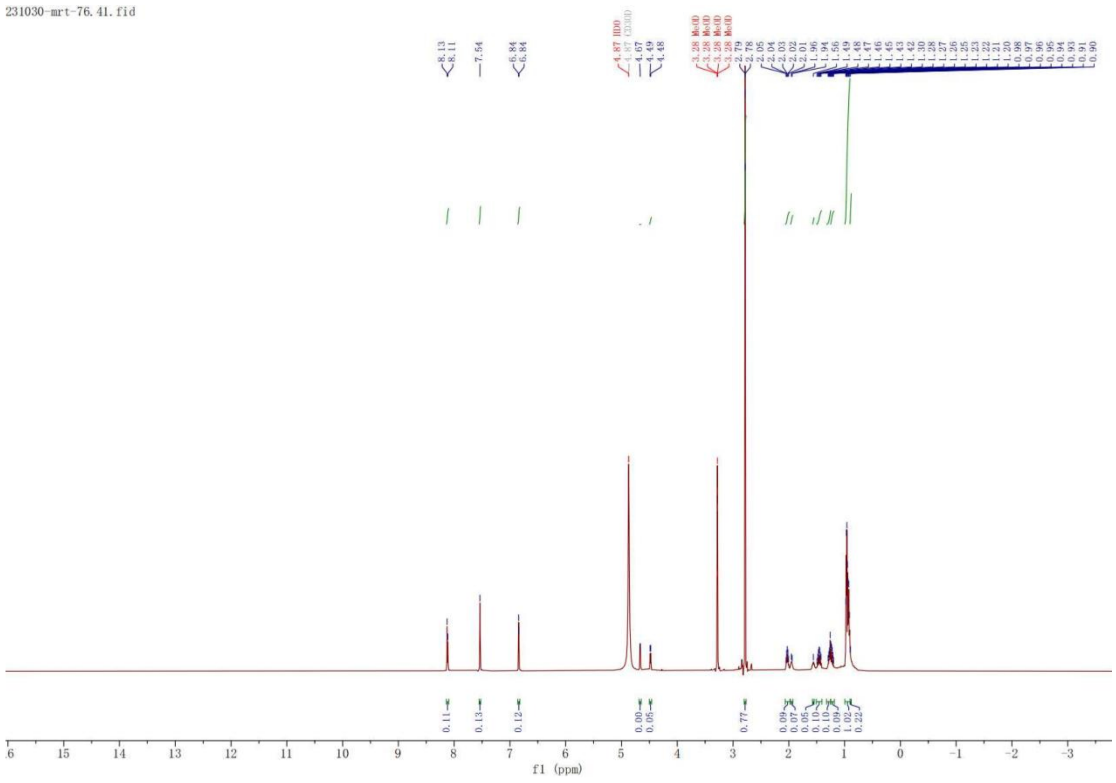

¹³C NMR of compound 2e

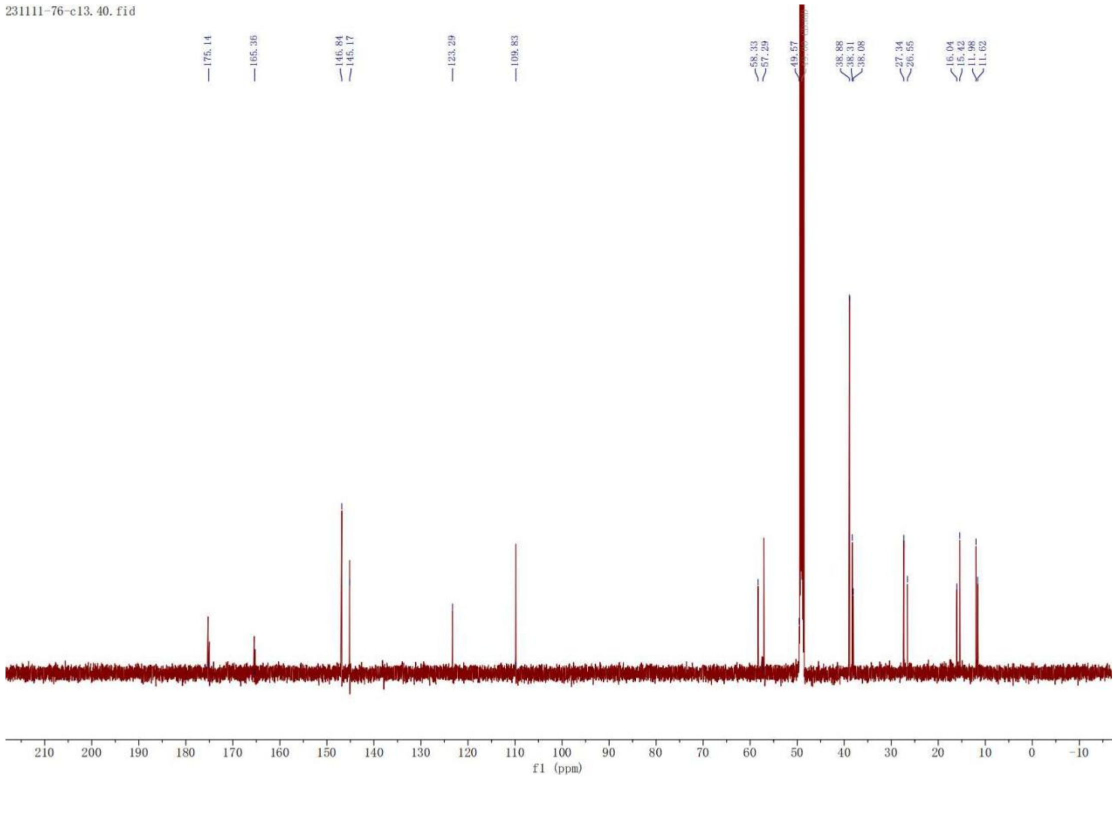

^{1}H NMR of compound 2f

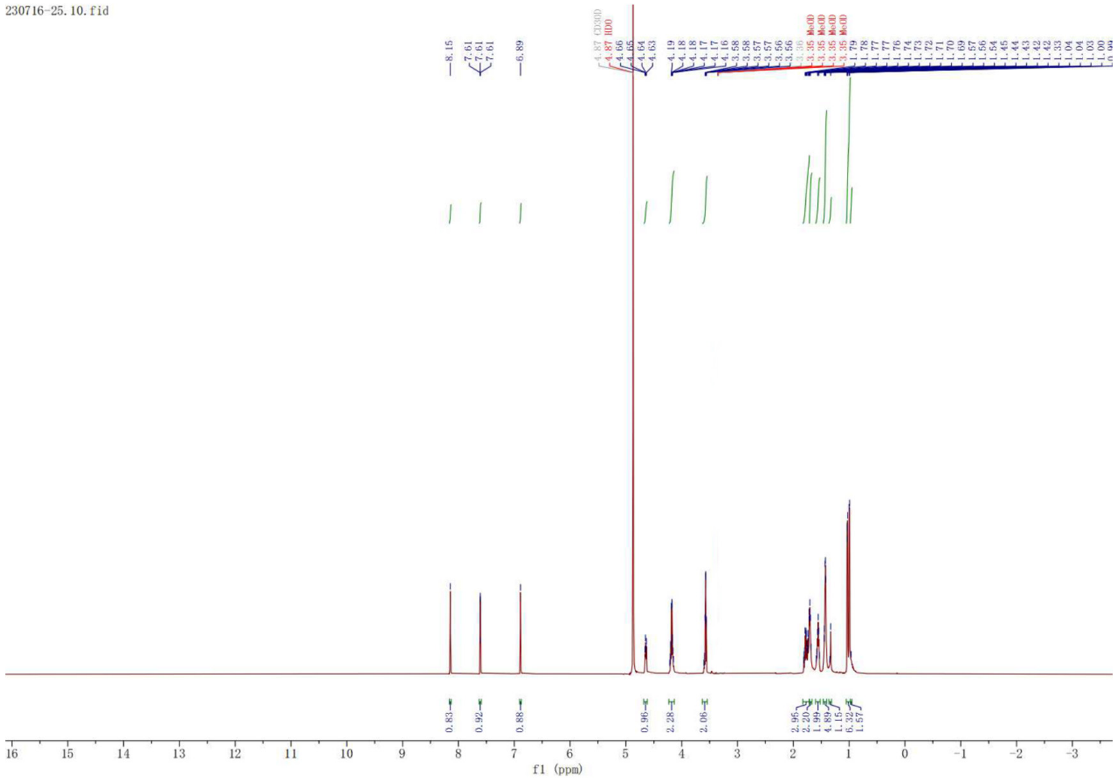

^{1}H NMR of compound 3a

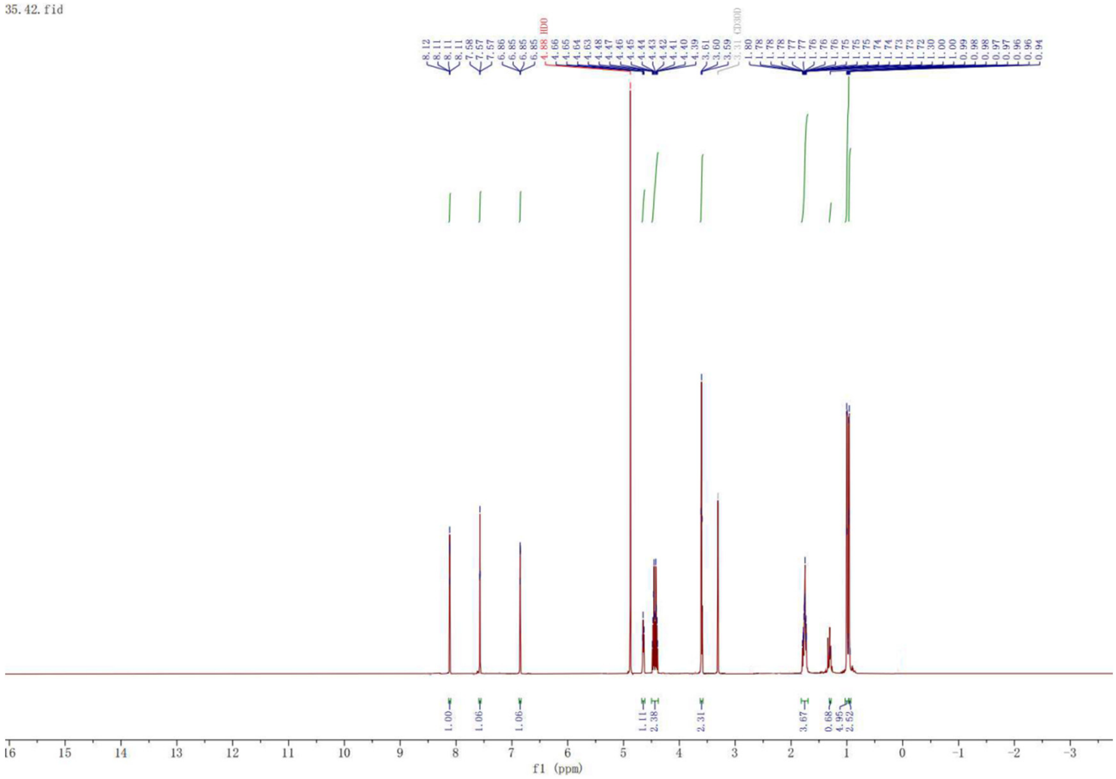

^{13}C NMR of compound 3a

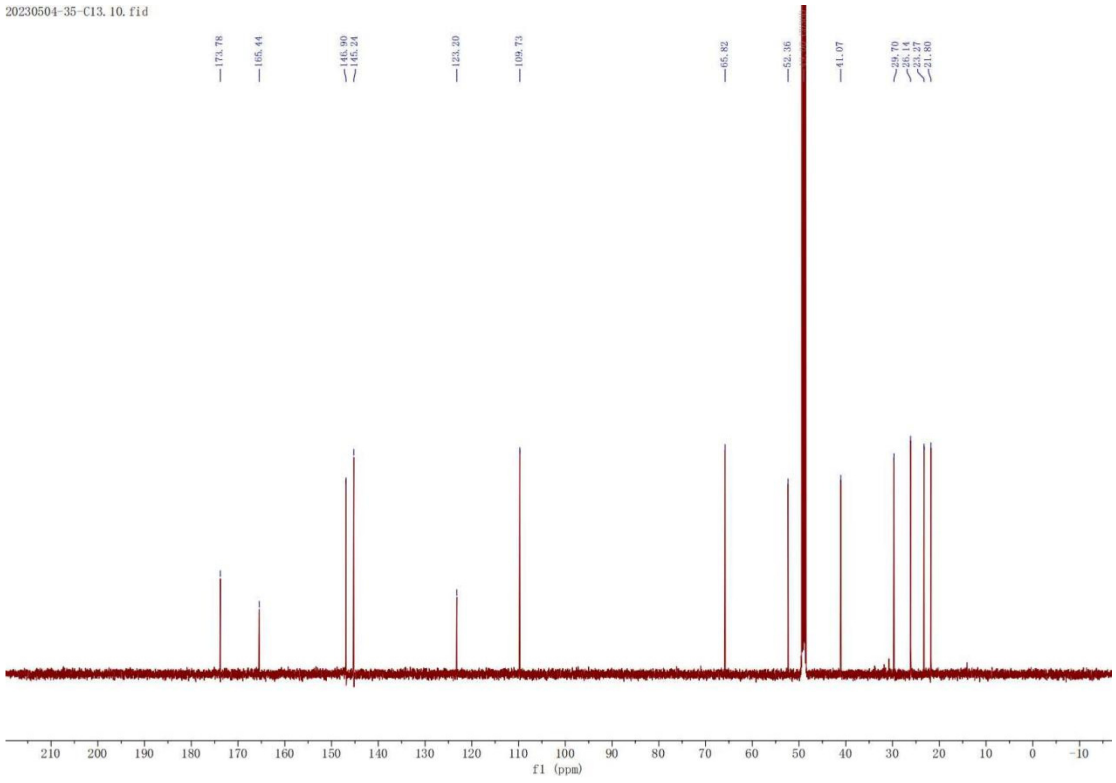

^{1}H NMR of compound 3b

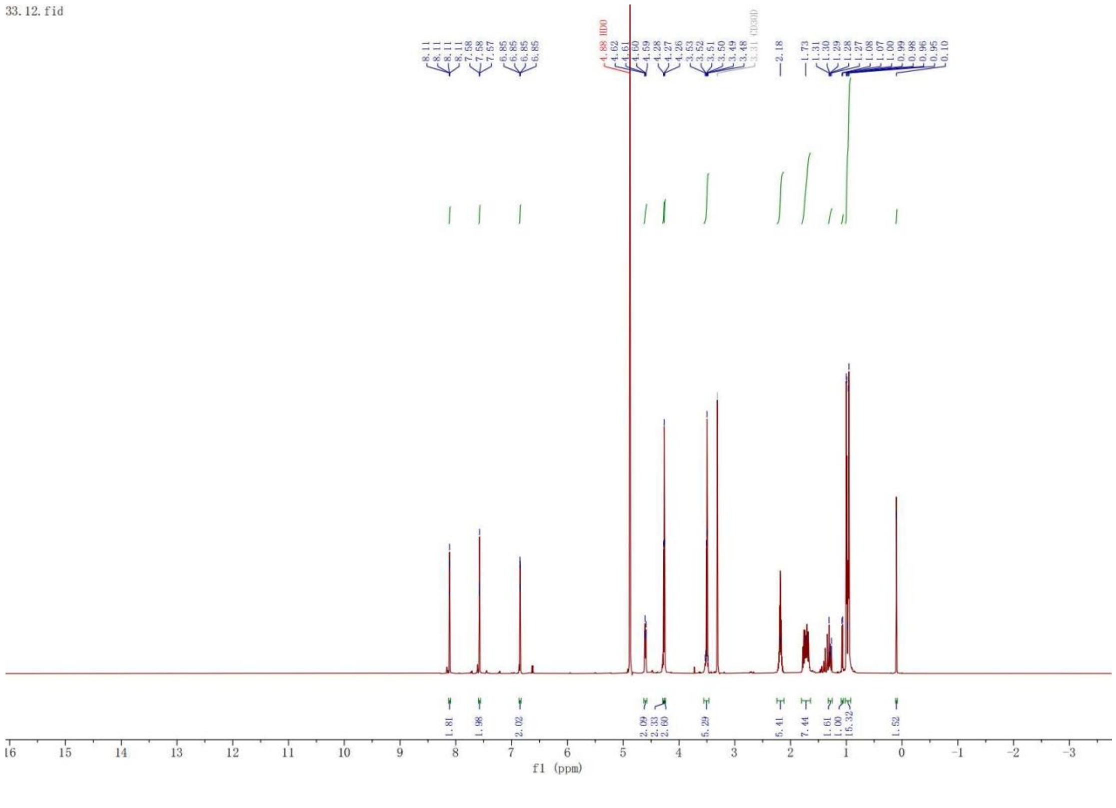

^{13}C NMR of compound 3b

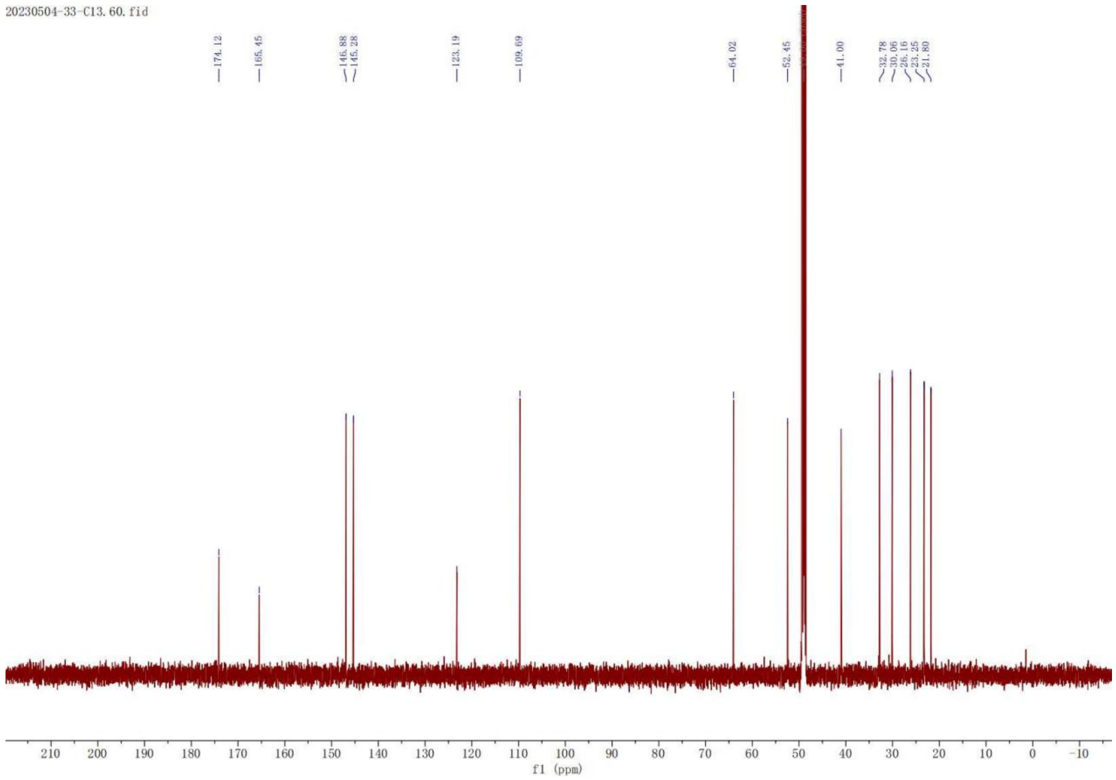

^{1}H NMR of compound 3c

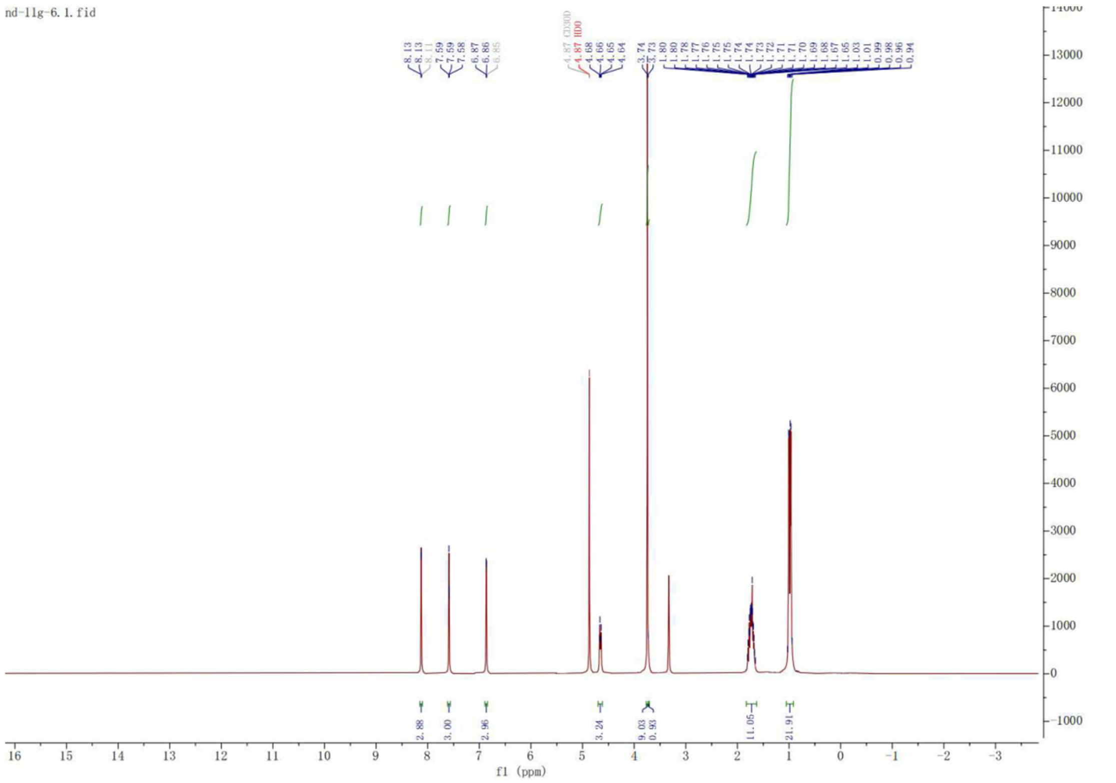

^{1}H NMR of compound 3d

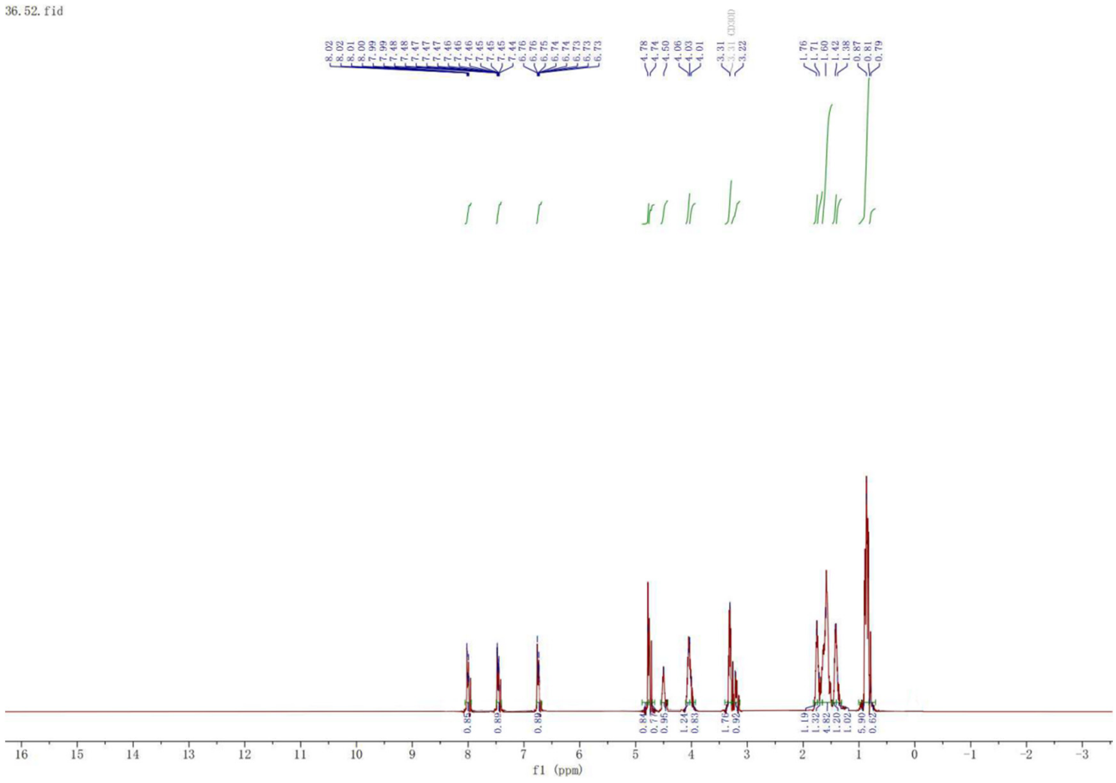

^{13}C NMR of compound 3d

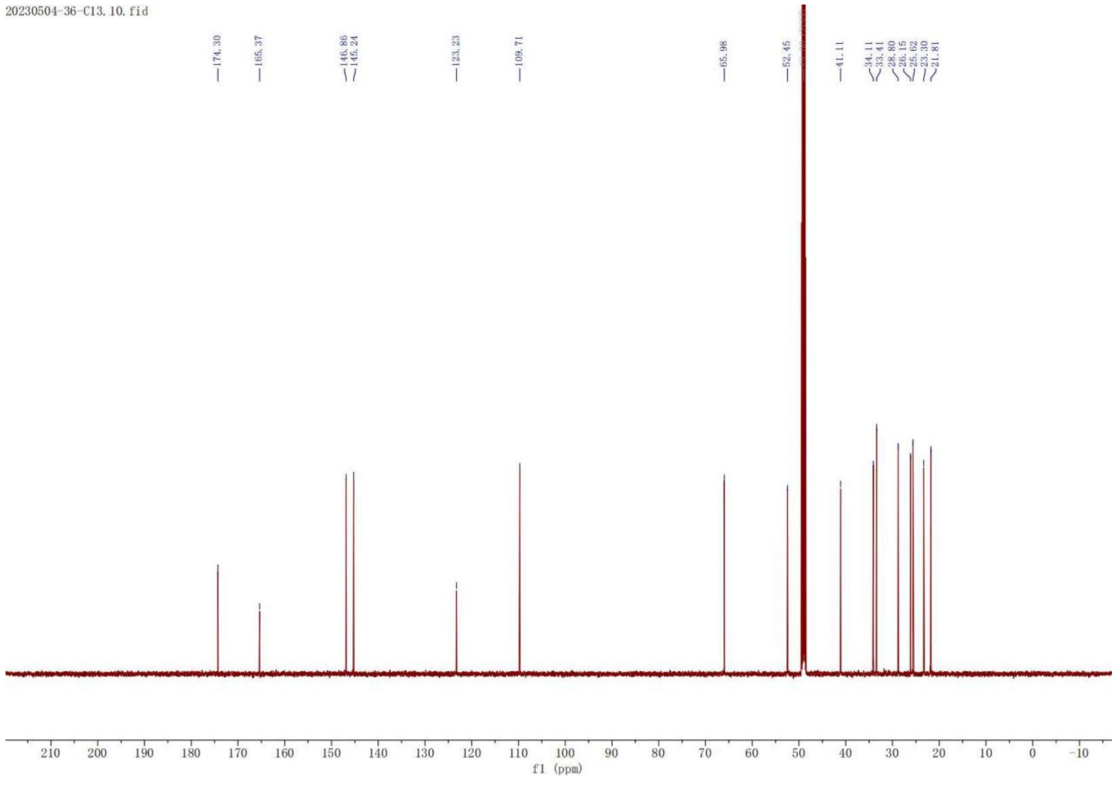

^{1}H NMR of compound 3e

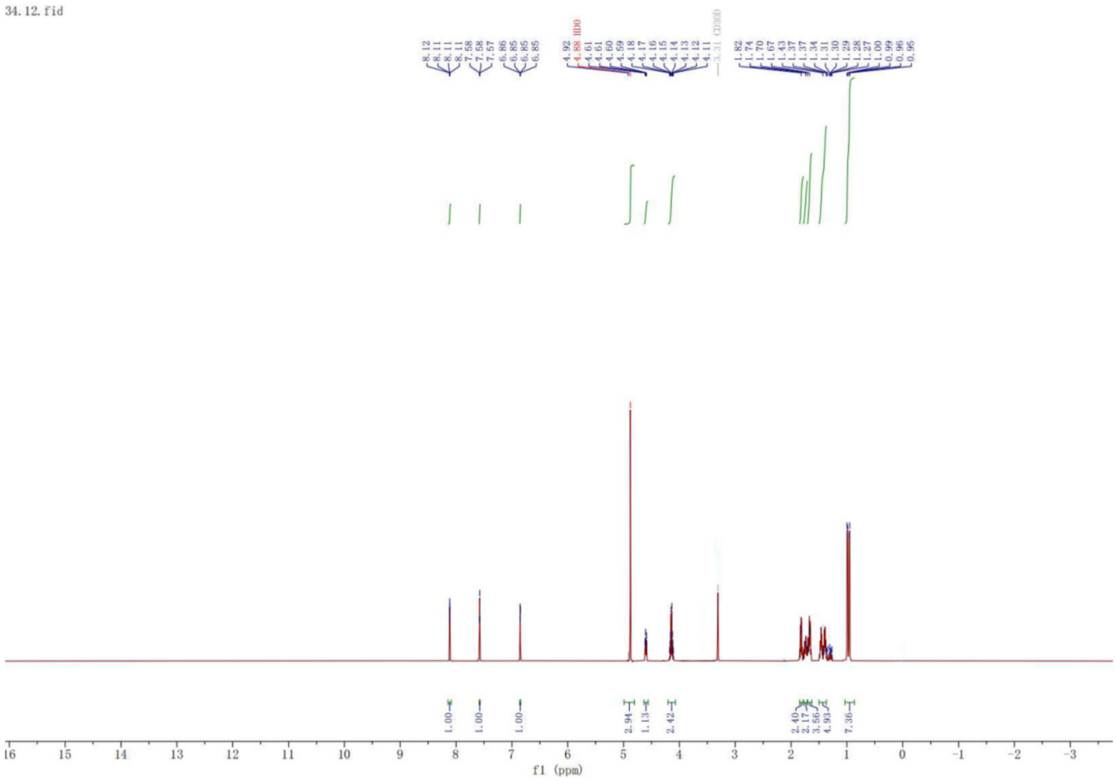

^{13}C NMR of compound 3e

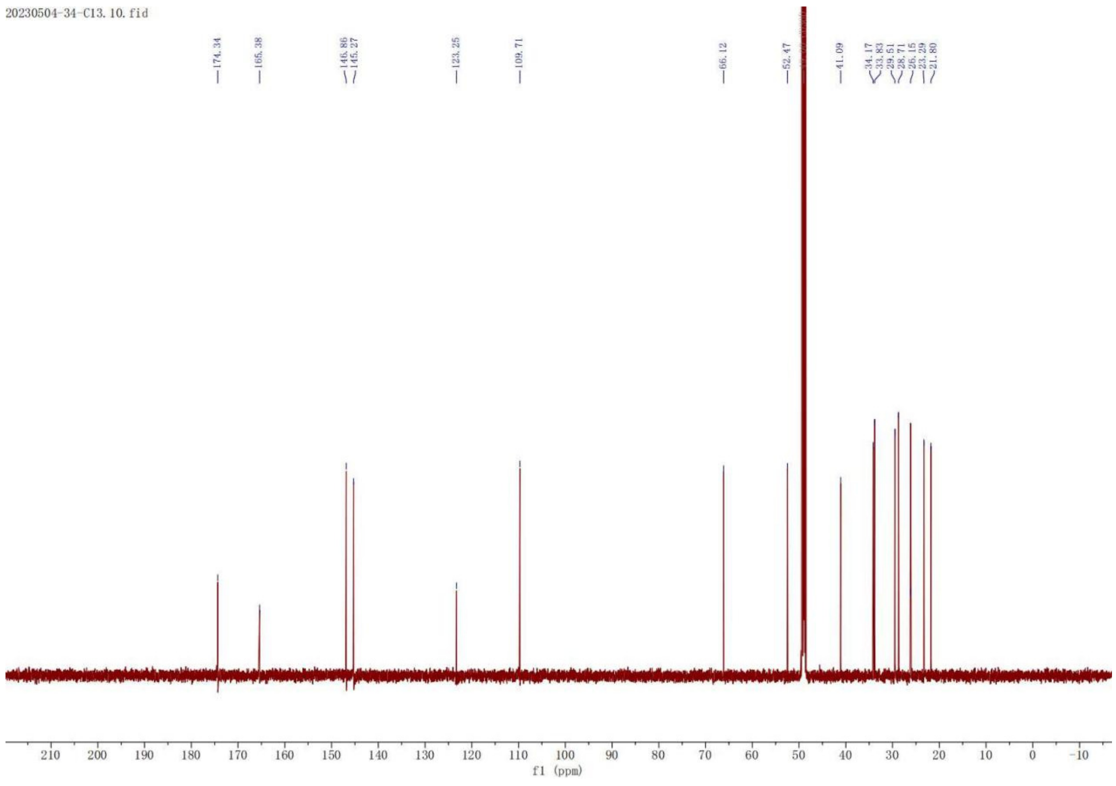

^{1}H NMR of compound 3f

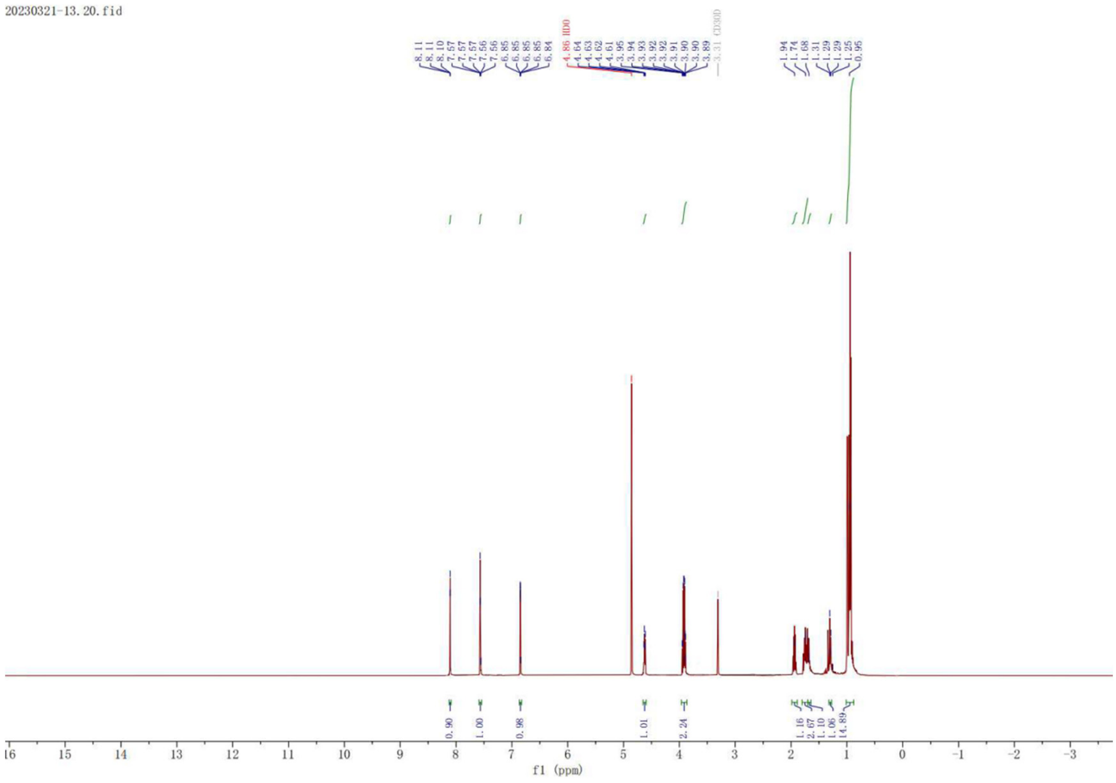

^{13}C NMR of compound 3f

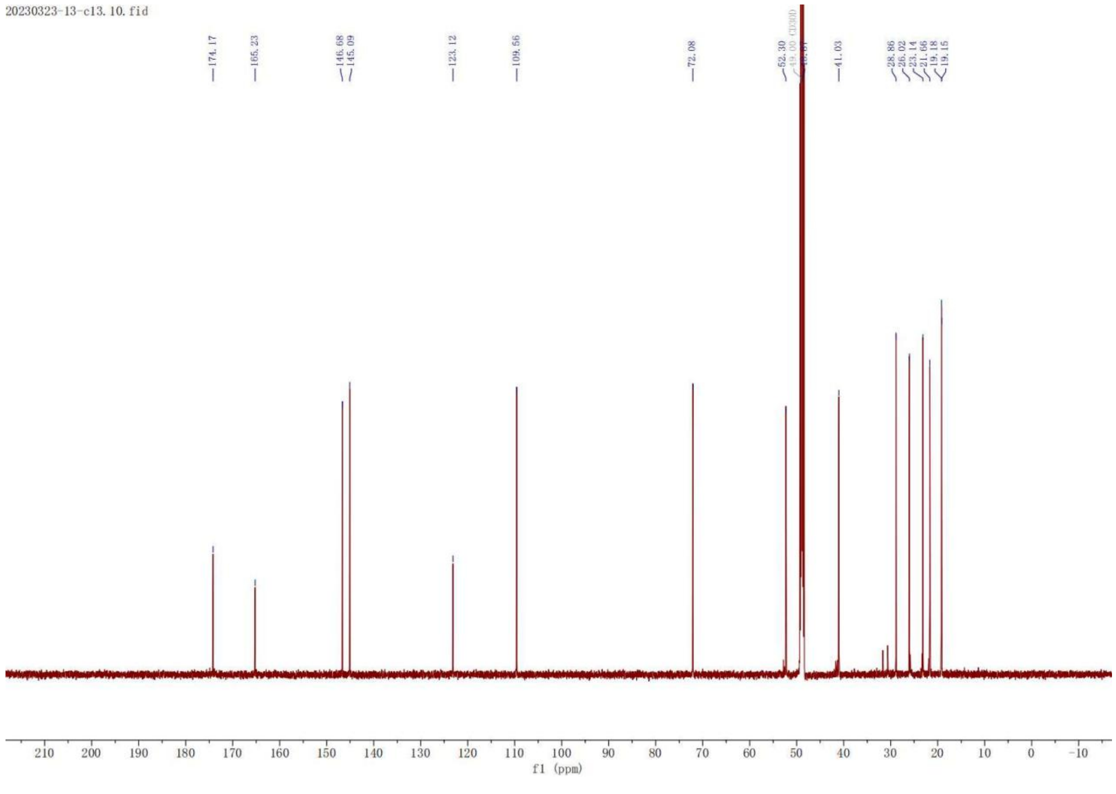

^{1}H NMR of compound 3g

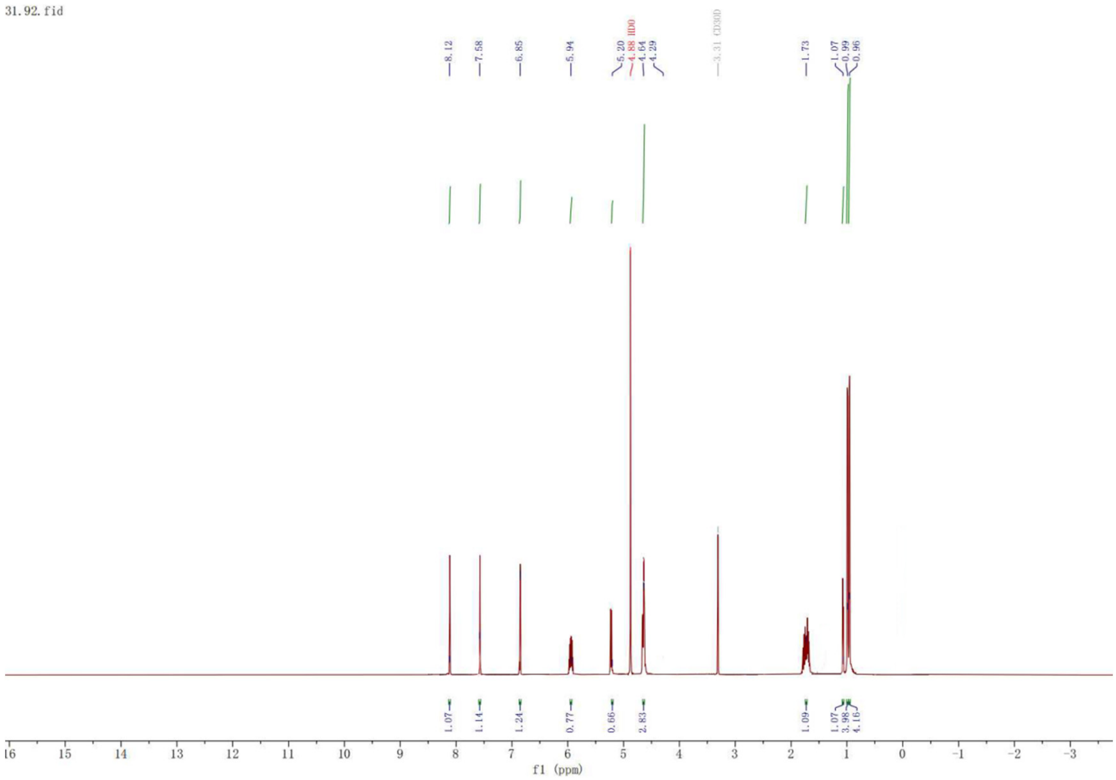

^{1}H NMR of compound 3h

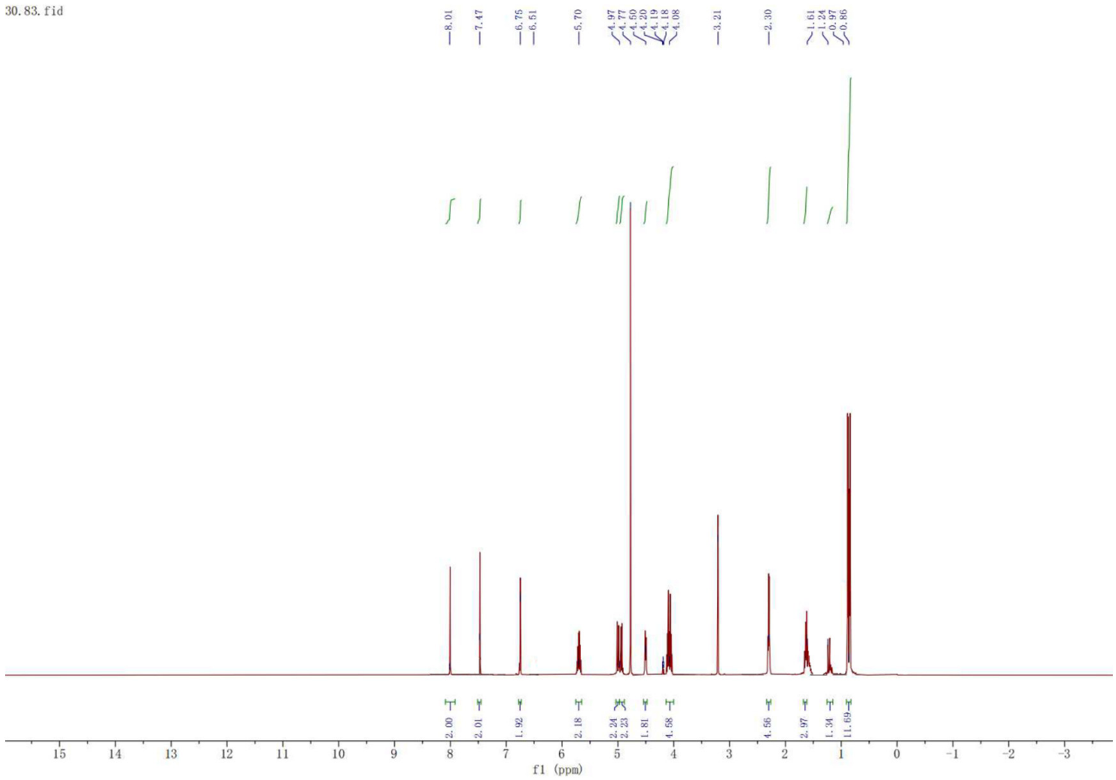

^{13}C NMR of compound 3h

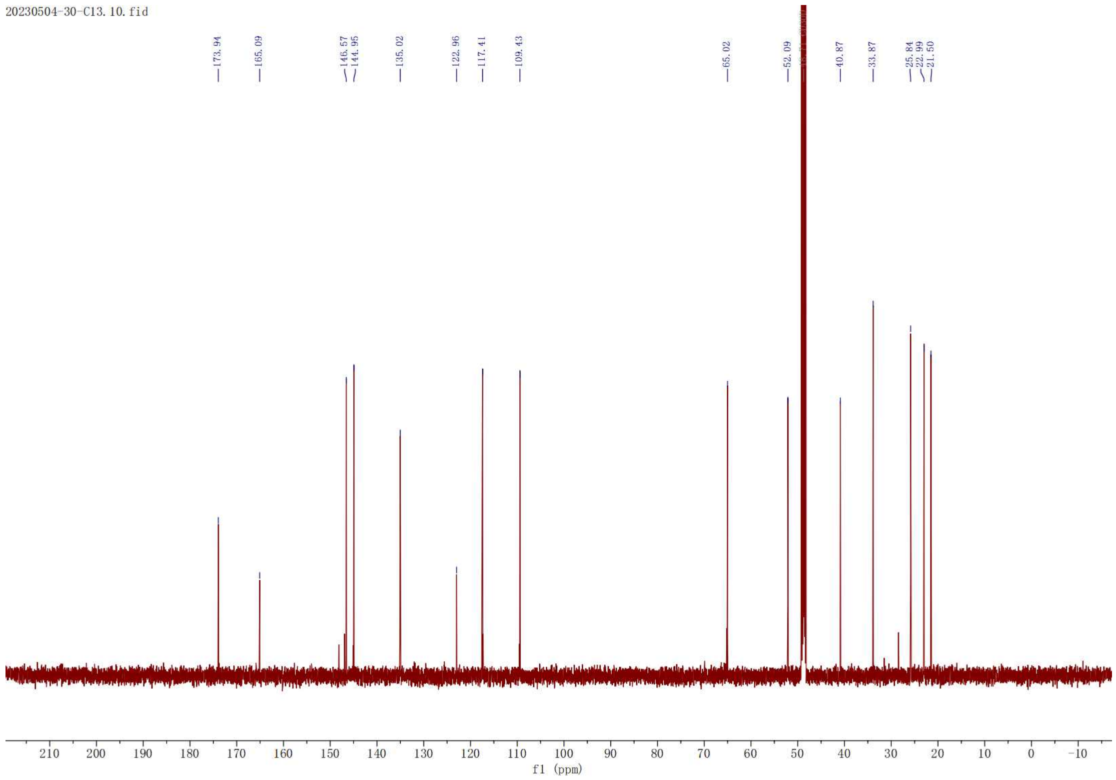

^{1}H NMR of compound 3i

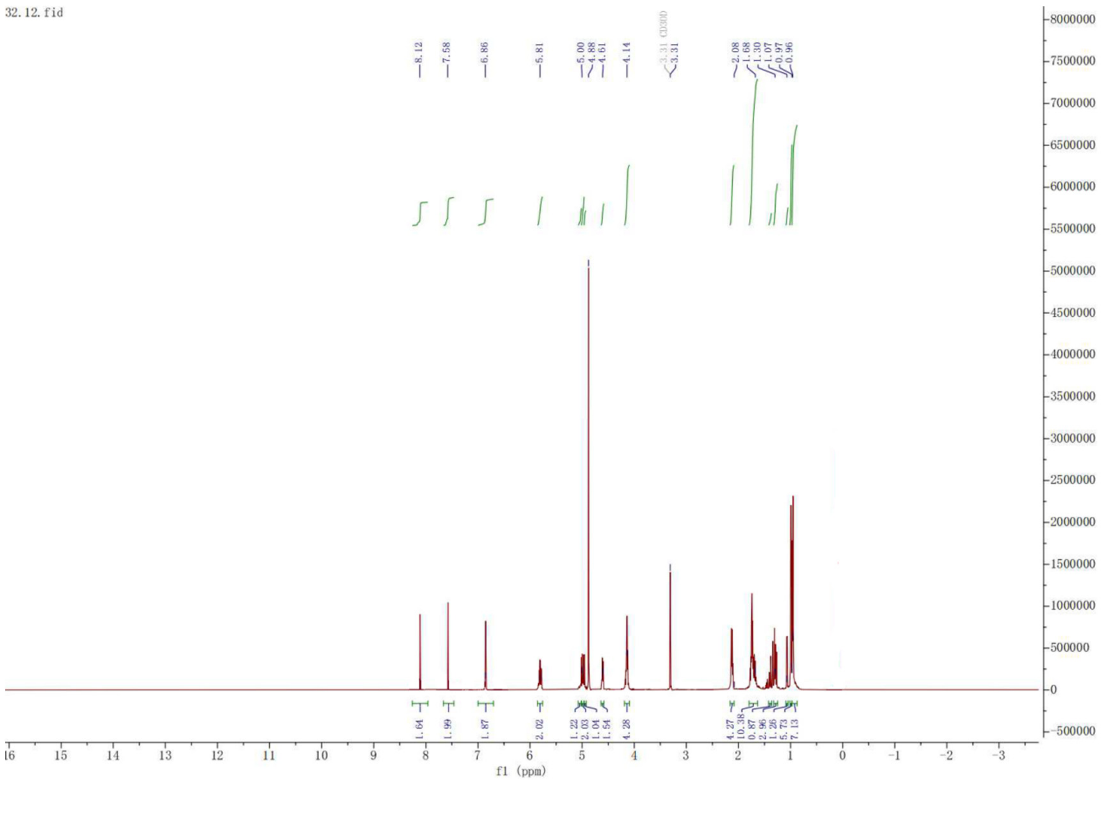

^{13}C NMR of compound 3i

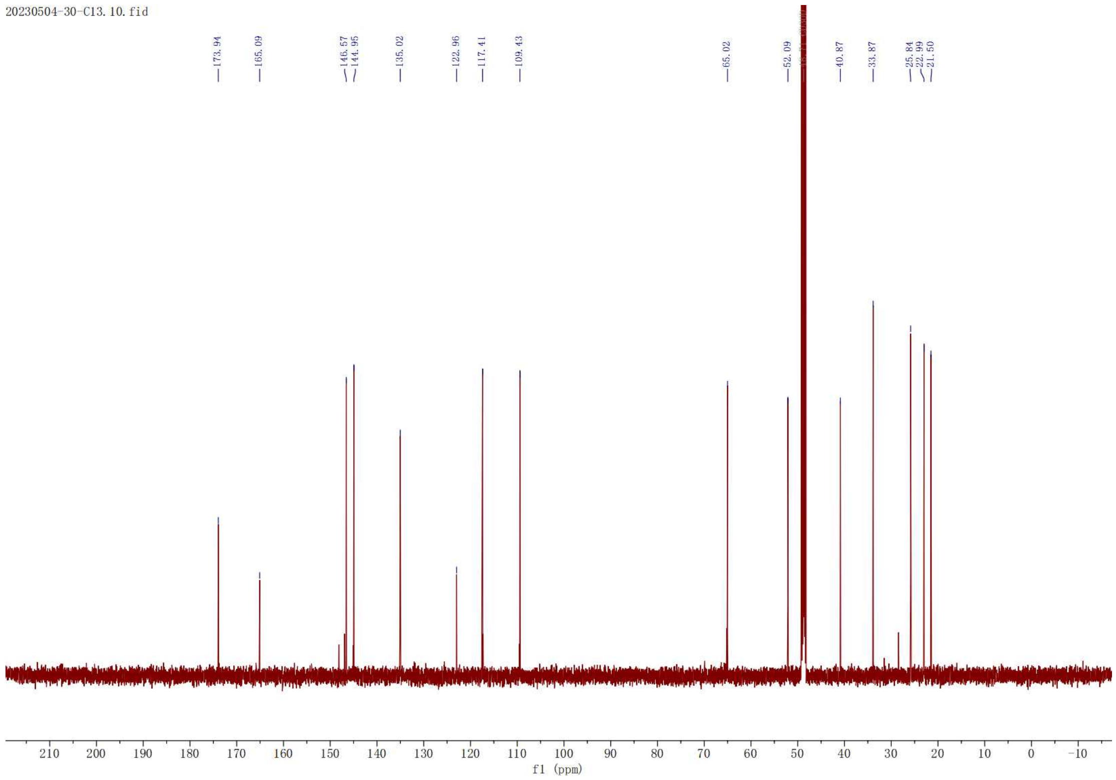

^{1}H NMR of compound 3j

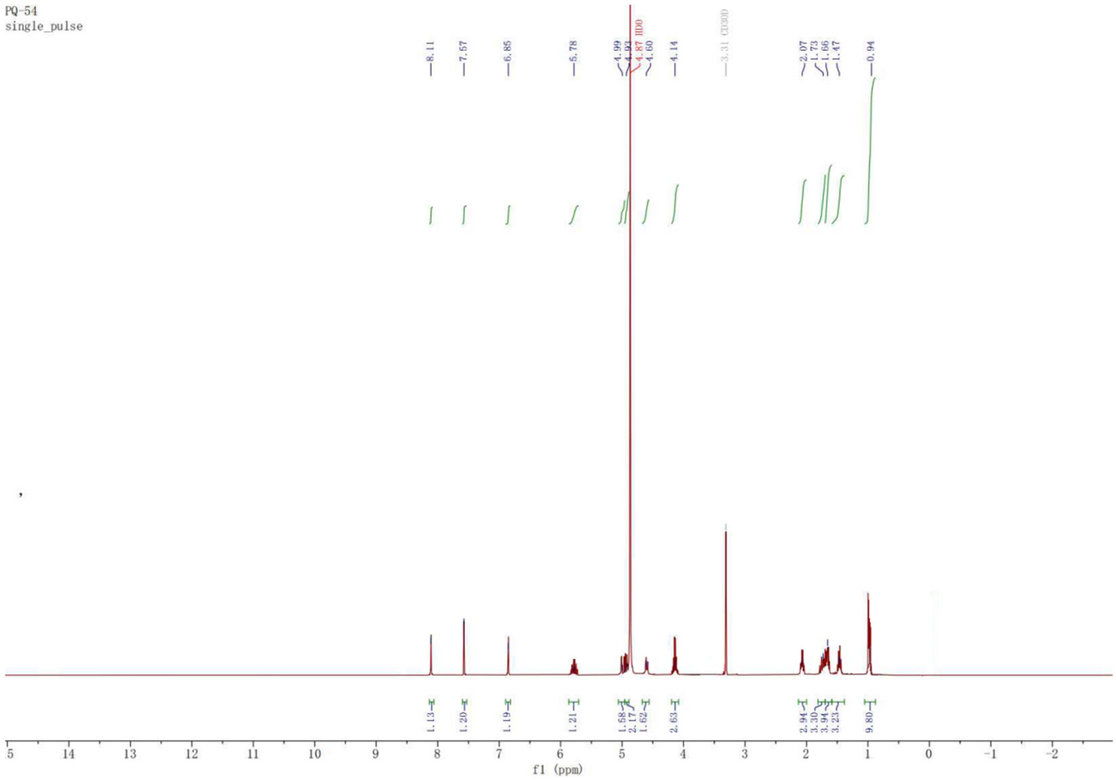

^{13}C NMR of compound 3j

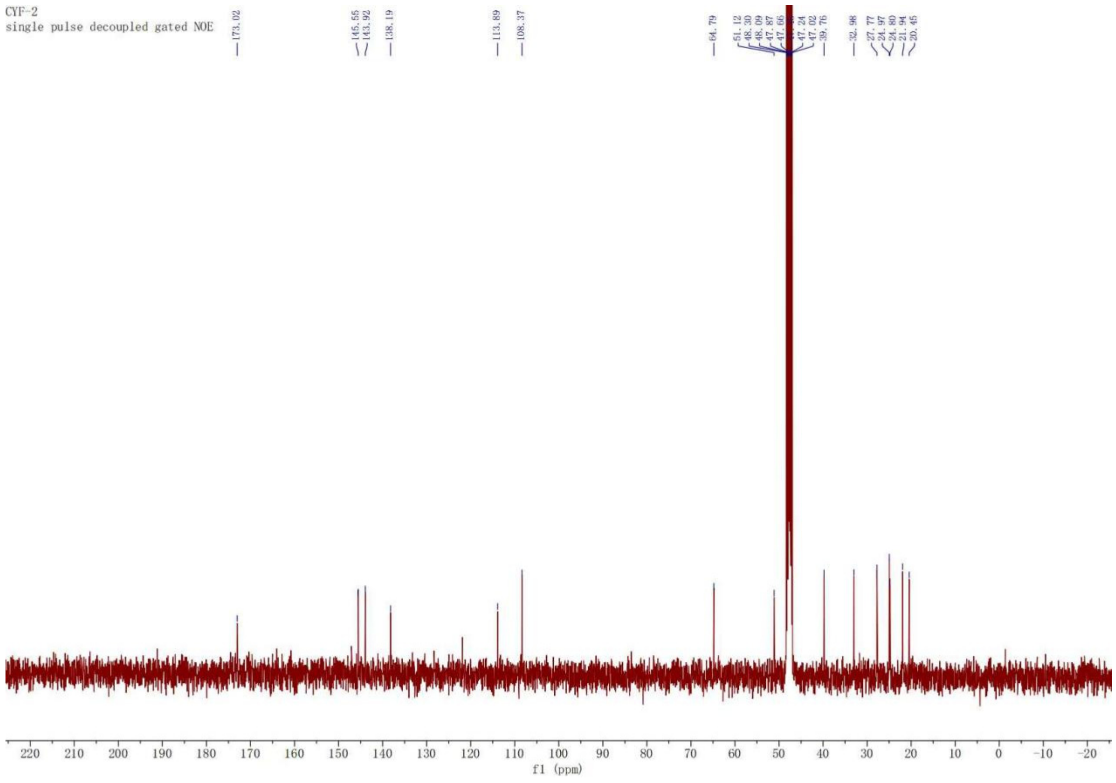

^{1}H NMR of compound 3k

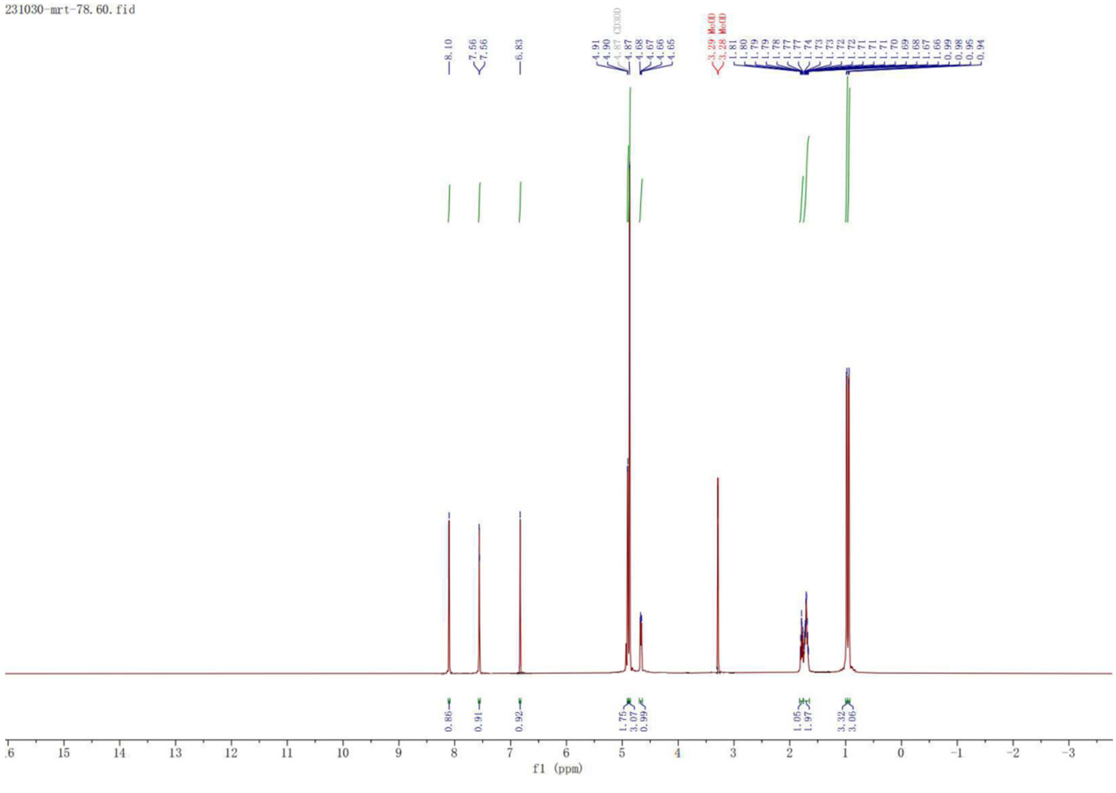

^{13}C NMR of compound 3k

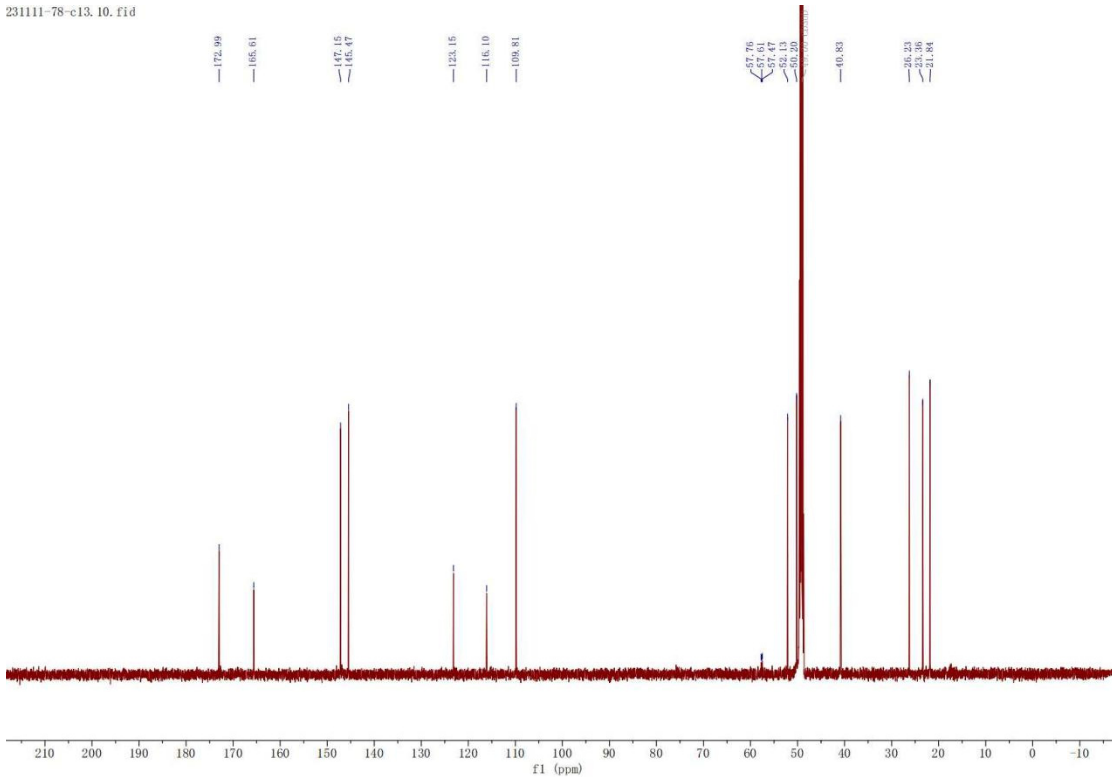

^{1}H NMR of compound 4a

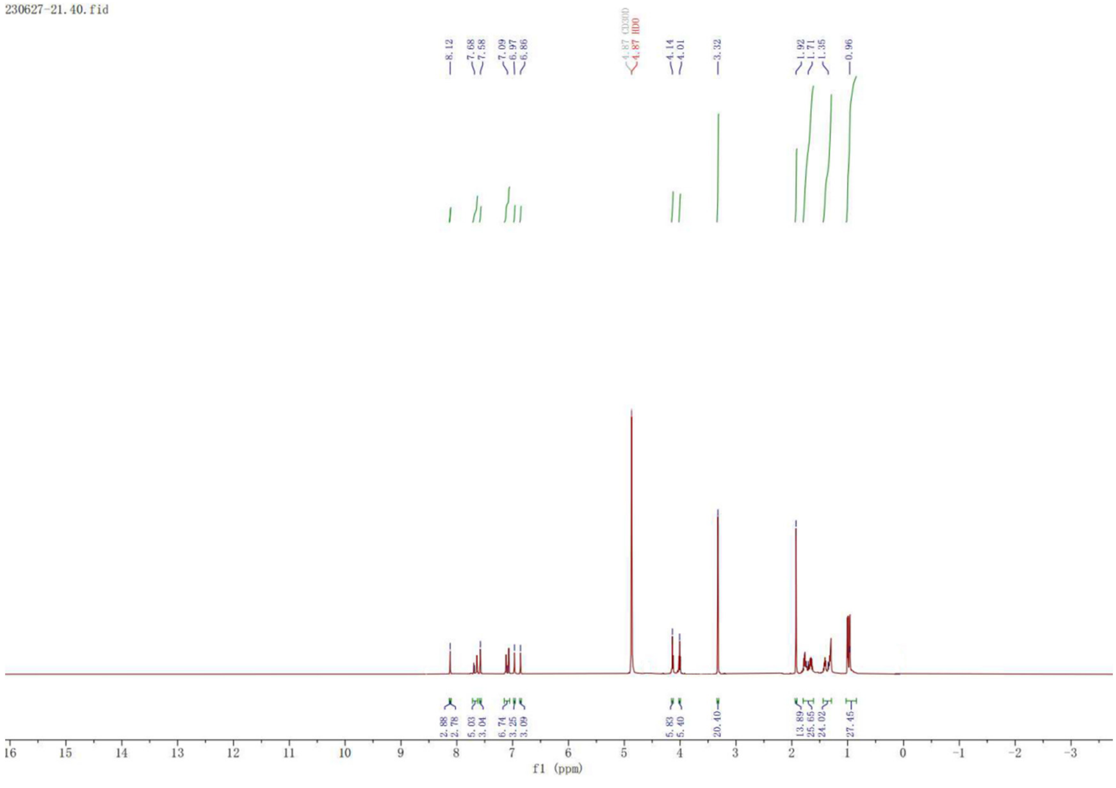

^{1}H NMR of compound 4b

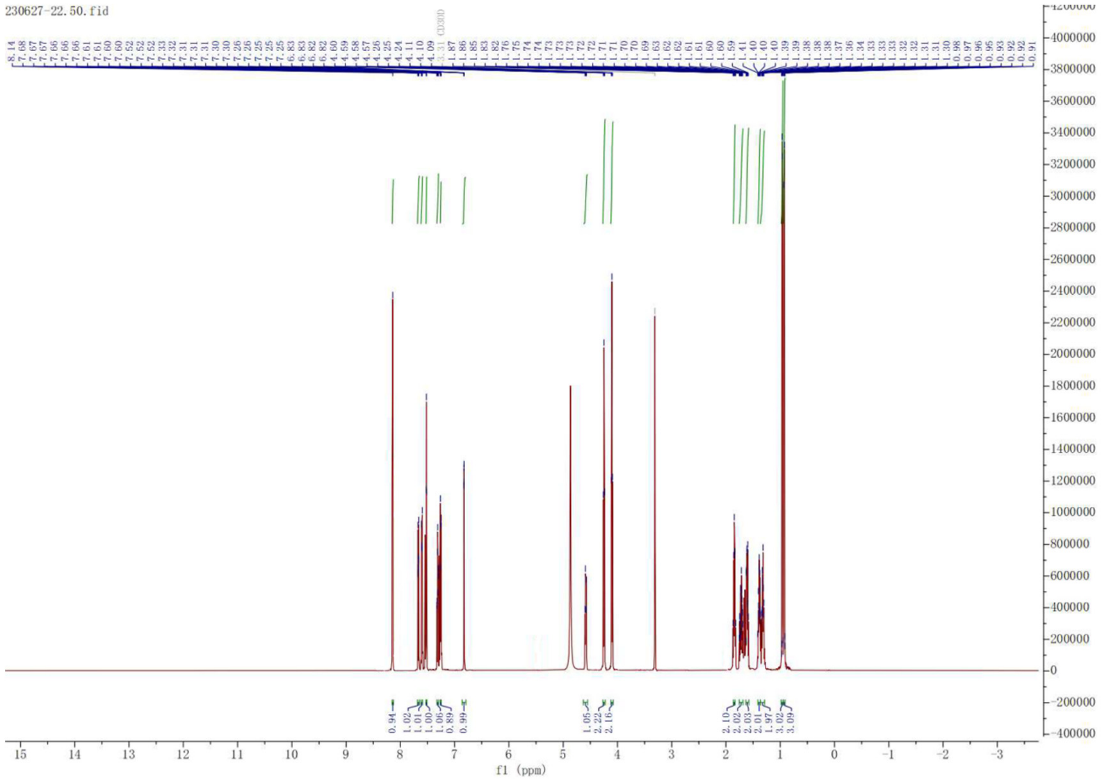

^{1}H NMR of compound 4c

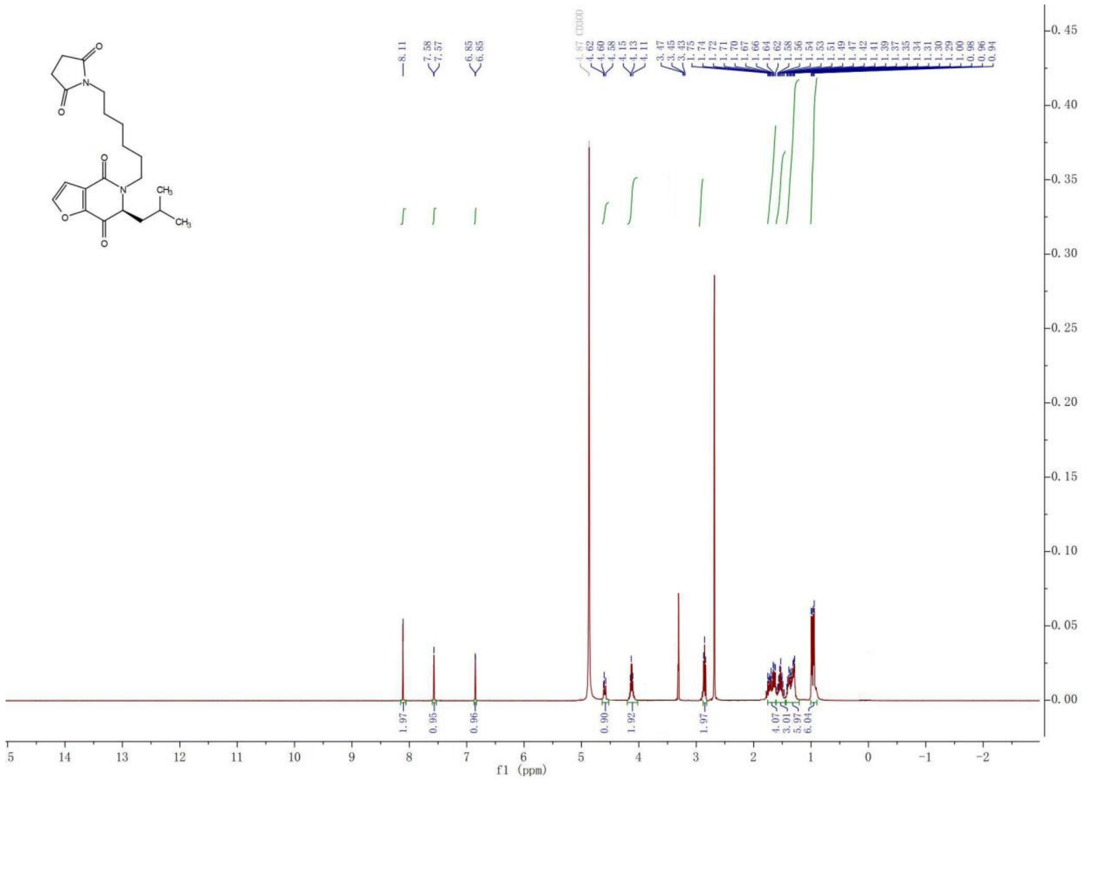

^{13}C NMR of compound 4c

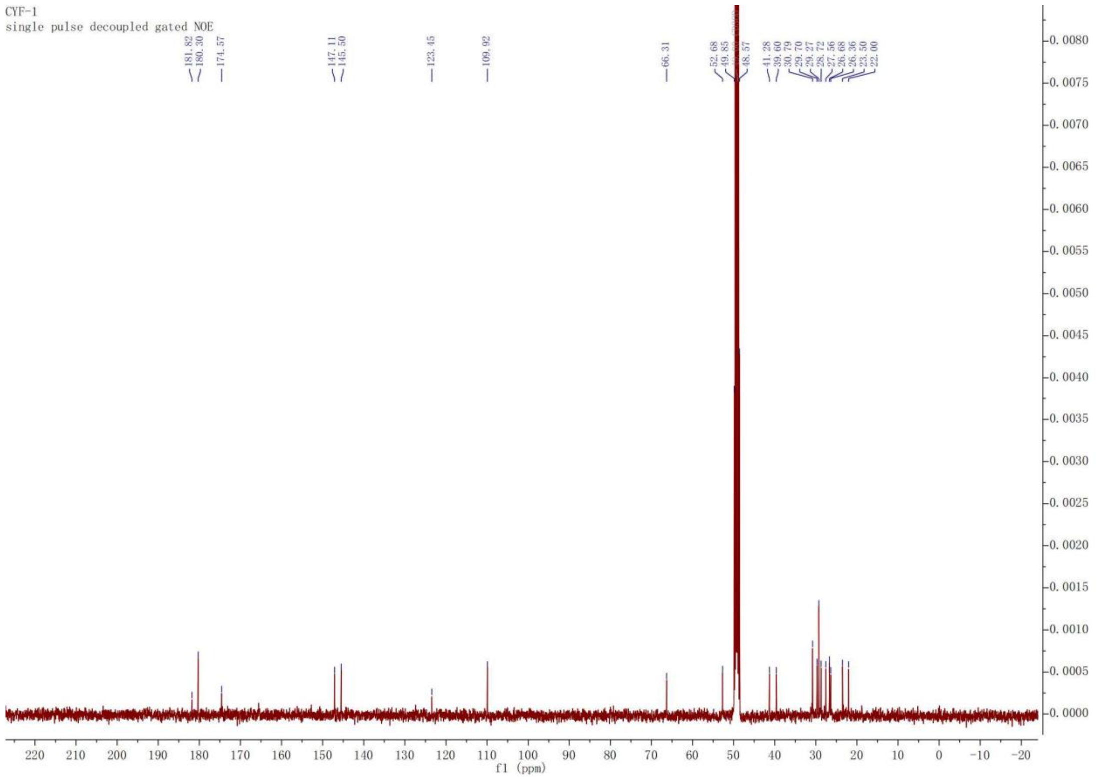

^{1}H NMR of compound 4d

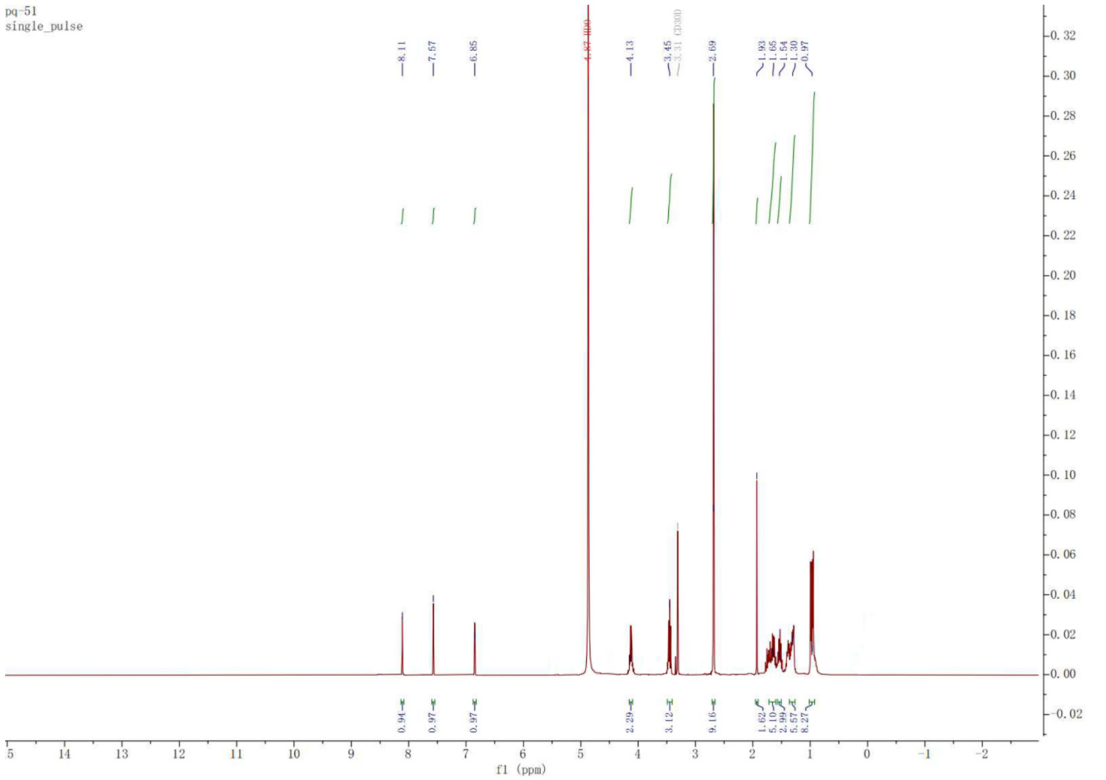

^{13}C NMR of compound 4d

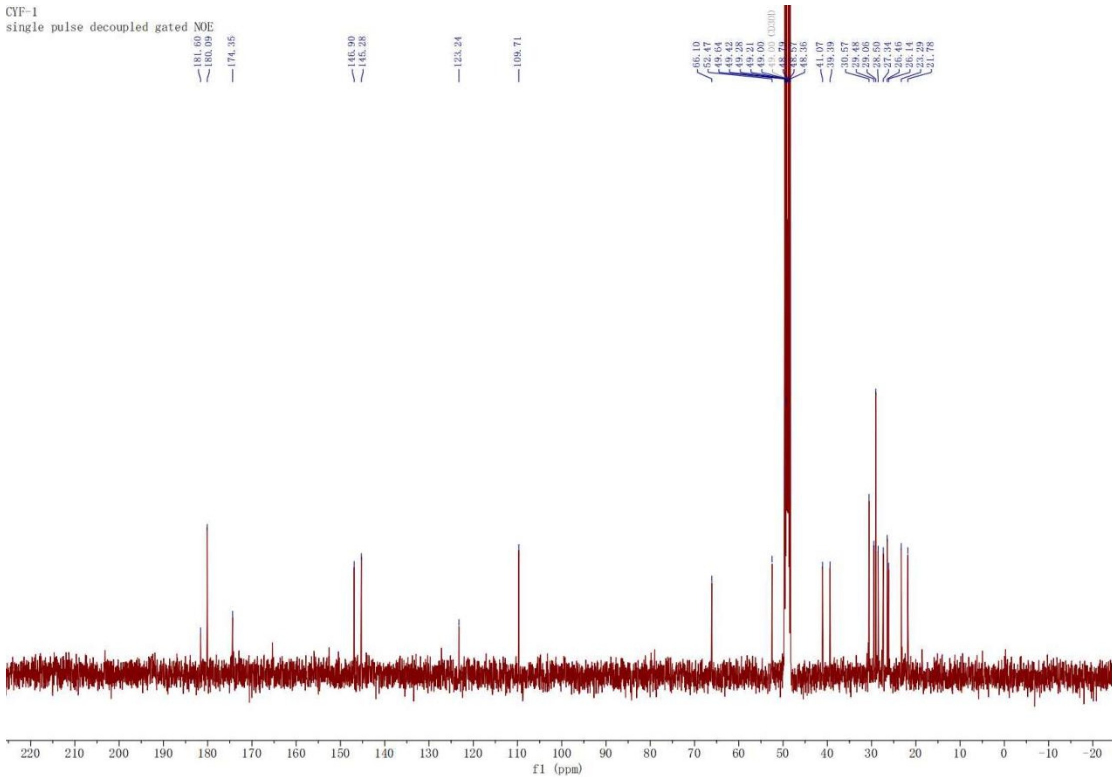

Supplement: Supplementary file 1 [file ijms-25-09634-s001.zip › ijms-3125728-supplementary.pdf]
